# Blood-Count-Derived Inflammatory Biomarkers and Characterization of Super-Responder Profile in Psoriatic Patients Receiving Biological Treatment: A Single-Center Study

**DOI:** 10.3390/ijms262110770

**Published:** 2025-11-05

**Authors:** Agnieszka Hołdrowicz, Radosław Zajdel, Agnieszka Żebrowska

**Affiliations:** 1 Department of Dermatology and Venereology, Medical University of Lodz, 90-647 Lodz, Poland; 2 Department of Economic and Medical Informatics, University of Lodz, 90-214 Lodz, Poland; 3 Department of AI in Healthcare, Medical University of Lodz, 90-645 Lodz, Poland

**Keywords:** psoriasis, super-response, biological treatment, non-specific inflammatory markers

## Abstract

In recent years, monoclonal antibodies targeting key cytokines underlying the occurrence of psoriatic skin lesions and joint involvement, i.e., Tumor Necrosis Factor-alpha (TNF-α), Interleukin 17 (IL-17), Interleukin 12 (IL-12), and Interleukin 23 (IL-23), have become more commonly used in the therapy of psoriasis. Due to the high effectiveness, a favorable safety profile, and growing availability of biological treatment methods, the number of patients receiving chronic monoclonal antibody therapy is increasing each year. However, the factors affecting the effectiveness of biological drugs are not fully recognized. The study aimed at analyzing the clinical profile of patients and non-specific inflammatory markers in terms of the response to the psoriasis treatment with IL-17, IL-23, IL-12/23, and TNF-α inhibitors. The analysis involved 185 patients receiving biological therapy in the Department of Dermatology and Venereology at the Medical University of Lodz, which resulted in a total of 222 treatment cycles (TC). The super-response was defined as 100% reduction in the Psoriasis Area and Severity Index (PASI 100), at week 16 (±4 weeks) of therapy. Our study indicates that the chance of achieving a super-response was higher among younger patients with no psoriatic lesions on palms and soles, not suffering from non-alcoholic fatty liver disease, previously treated with methotrexate, and characterized by a higher level of derived Neutrophil-to-Lymphocyte Ratio (dNLR) at the beginning of treatment.

## 1. Introduction

Psoriasis (PsO) is an autoimmune disease caused by chronic inflammation. The condition is increasingly recognized as a systemic disease associated with a higher risk of cardiovascular disorders, metabolic syndrome, depression, inflammatory bowel disease, non-alcoholic fatty liver disease, and uveitis. Over 30% of psoriatic patients also suffer from joint involvement leading to deformations and destruction of joints [1,2,3,4]. In recent years, monoclonal antibodies targeting key cytokines underlying the occurrence of psoriatic skin lesions and joint involvement, i.e., Tumor Necrosis Factor-alpha (TNF-α), Interleukin 17 (IL-17), Interleukin 12 (IL-12), and Interleukin 23 (IL-23), have become more commonly used in the therapy of psoriasis [5].

Due to the high effectiveness, a favorable safety profile, and growing availability of biological treatment methods, the number of patients receiving chronic monoclonal antibody therapy is increasing every year. However, the factors affecting the effectiveness of biological drugs are not fully recognized [6,7,8,9,10,11,12]. The literature on the subject analyzes the phenomenon of a super-response to treatment with monoclonal antibodies, regarded as near or complete clearance of the skin within a short period after exposure to the therapy. However, no precise definition of this response is provided, and it varies across published studies [6,7,8,9,10,11,12]. Super-response is most often associated with a Psoriasis Area and Severity Index 100 (PASI100) response to the therapy, i.e., complete clearance of the skin, within 12 to 28 weeks [6]. Rompoti et al. define a super-response as achieving Psoriasis Area and Severity Index (PASI) ≤ 1 at weeks 12 and 16 [7], while Feldman et al. as reaching 90% reduction in the Psoriasis Area and Severity Index (PASI90) at week 28 [8]. Some studies define it as both obtaining and maintaining positive clinical results in terms of skin lesions [9,10,11]. Loft et al. describe super-response as achieving and maintaining PASI < 3 skin lesion severity for a period of six to 60 months in the group of bio-naïve patients treated with a single biological drug for at least five years [9]. Similarly, a target defined in two other studies is maintaining PASI100 response for 88–100 [10] or 104 [11] weeks, respectively. The Early Super Response is also described in the literature as achieving PASI100 at week 4 of therapy [12]. Moreover, using the absolute rather than the relative PASI score appears to be of greater value due to the easier translation of research outcomes into everyday clinical practice [13]. Regardless of assumptions and definitions, the objective of currently conducted studies is to identify factors that influence the response to biological drugs, enabling future personalization and reducing the costs of biological treatment methods in the therapy of psoriasis.

The study aimed at analyzing the clinical profile of patients and non-specific inflammatory markers in terms of the response to the psoriasis treatment with IL-17, IL-23, IL-12/23, and TNF-α inhibitors.

## 2. Results

The analysis involved 185 patients receiving a biological therapy within the B.47 drug program of the Ministry of Health of the Republic of Poland. Some of the patients were treated with more than one biological drug, which resulted in a total of 222 treatment cycles (TC). In the study group, a super-response was observed in 94 patients, accounting for 42.34% of TC. Two patients achieved a super-response twice; in both cases, the therapy was modified due to the secondary treatment failure. Considering treatment cycles, men constituted the majority of the study group (123 TC/55.41%). The average age of patients at the beginning of treatment was 44 ± 14.74 years (super-responders (SR) 40.70 ± 13.38 years vs. non-super-responders (nSR) 46.42 ± 15.16 years), and the average PASI score was 17.57 ± 8.16. A family medical history of psoriasis was reported in 47.06% of all TC and more frequently observed in the super-responder group (SR: 52.69% vs. nSR: 42.97%); however, this observation was not statistically significant (*p*-value > 0.05). Clinical data of the study group are presented in Table 1.

No statistically significant correlations were found between sex, body weight, BMI value, age of disease onset, disease duration before the initiation of biological treatment, initial severity of the skin lesions with PASI and BSA (Body Surface Area) scores at the beginning of the therapy, baseline assessment of quality of life using DLQI (Dermatology Life Quality Index) scale and achieving a super-response at week 16 (±4) of the treatment. However, an older age was associated with a lower chance of a super-response, and with every one-year increase in age, the odds of achieving a super-response decreased by approximately 3.3% (Odds Ratio (OR) = 0.967; 95%Confidence Interval (CI): 0.947–0.988; *p* = 0.002).

Treatment cycles with IL-23 inhibitors amounted to 51.35% of all cases (risankizumab 71 TC/31.98%, guselkumab 32 TC/14.41%, tildrakizumab 11 TC/4.95%). Monoclonal antibodies targeting IL-17A constituted 26.58% (sekukinumab 37 TC/16.67%, iksekizumab 22 TC/9.9%) and bimekizumab aiming against IL-17A/F 10.36% (23 TC). TNF-α inhibitors comprised 9.01% (adalimumab 18 TC/8.11%), infliximab 2 TC/0.9%) of the analyzed treatment cycles. Ustekinumab, an inhibitor of IL-12/23, with 6 TC accounted for 2.7% of cases. In the study group, 152 treatment cycles were applied to bio-naïve patients representing 68.47% of all TC. No statistically significant association was found between the number of previously received biological drugs and a super-response.

The highest percentage of SR was observed among the patients treated with infliximab (SR: 100% vs. nSR 0%), followed by bimekizumab (SR: 91.30% vs. nSR: 8.70%) and ixekizumab (SR: 59.09% vs. nSR: 40.91%). In the group of patients receiving IL-23 inhibitors, a super-response was more often reported in those undergoing risankizumab therapy (SR: 39.44% vs. nSR: 60.56%) but was comparable to individuals receiving guselkumab therapy (SR: 34.38% vs. nSR: 65.63%). The lowest percentage of SR was observed in the group treated with tildrakizumab (SR: 9.09% vs. nSR: 90.91%), followed by ustekinumab (SR: 16.67% vs. nSR: 83.33%) and adalimumab (SR: 33.33% vs. nSR: 66.67%).

On the basis of multinomial logistic regression analysis, it was determined that ixekizumab and bimekizumab therapy were associated with, respectively, a 14-fold (OR = 14.444; 95% CI: 1.562–133.586; *p* = 0.0186) and 105-fold (OR = 105; 95% CI: 8.483–1299.596; *p* = 0.0003) higher odds of achieving a super-response, than the treatment with tildrakizumab. Clinical response to therapy after 24 weeks from the second time point was also assessed. Among the SR, such data were available for 75 of the 94 patients. Super-response was maintained in 82.67% of patients in this group. The highest percentage of SR was observed in the group treated with bimekizumab (100%), followed by sekukinumab (87.5%) and risankizumab (87%). No patient receiving TNF–α inhibitors sustained a PASI100 response at week 40 (±4 weeks). Tildrakizumab was excluded from this analysis due to an insufficiently large study group.

The analysis also included the number, type, and duration of previously received non-biologic therapies (methotrexate (MTX), ciclosporin, acitretin, Psoralen Ultra-Violet A (PUVA) therapy, Narrowband Ultraviolet B (NB-UVB) phototherapy).

A longer duration of methotrexate treatment was associated with a higher chance of a super-response, and with every one-month increase, the odds of achieving a super-response increased by approximately 2.1% (OR = 1.021; 95% CI: 1.002–1.040; *p* = 0.034). Methotrexate was administered orally or subcutaneously, in doses of 10 to 25 mg/week. The time between the discontinuation of methotrexate therapy and the initiation of biological treatment varied and did not always correspond to the washout period, which could have had an impact on the results obtained. No associations were found between the type or duration of other conventional methods applied before the initiation of biological treatment and achieving a PASI100 response at week 16 (±4 weeks) during monoclonal antibody therapy. The duration of treatment with respective conventional methods in the study group is presented in Table 2.

There was no statistically significant relationship between the duration of a biological therapy and SR or nSR group. However, it is worth mentioning that secondary treatment failure was more frequently observed in the nSR group (SR: 7.4% vs. nSR: 19.5%). The incidence rate of adverse reactions resulting in treatment termination amounted to 4.05% and was similar in both groups (SR: 4.3% vs. nSR: 3.9%). More than one adverse reaction was observed in 2.25% of patients.

### 2.1. Comorbidities

In the analyzed study group, 24.77% of patients suffered from psoriatic arthritis (PsA), which was more often reported in the SR group (SR: 27.66% vs. nSR: 22.66%); however, this disparity was not statistically significant. The study also analyzed co-occurrences of chronic kidney disease and cardiovascular, metabolic, thyroid, and depressive disorders. The incidence of comorbidities diagnosed in the study group is presented in Table 3. It was statistically confirmed that co-occurrence of non-alcoholic fatty liver disease was associated with a more than twofold reduction in the chance of achieving a super-response (OR = 0.443; 95% CI: 0.221–0.931; *p* = 0.032). No such relationships were determined for other comorbidities. There was also no statistically significant association between nicotinism and response to the biological therapy.

At the beginning of biological treatment, psoriatic skin lesions in special localizations (scalp, palms and soles, anogenital area, nails) were examined. Statistical analysis using a multinomial logistic regression model showed that the occurrence of psoriatic skin lesions on palms and soles was associated with an almost fivefold lower chance of achieving a super-response (OR = 0.222; 95% CI: 0.093–0.528; *p* = 0.001). No relationships were found for other special localizations.

### 2.2. Non-Specific Inflammatory Markers

**Figure 1:**
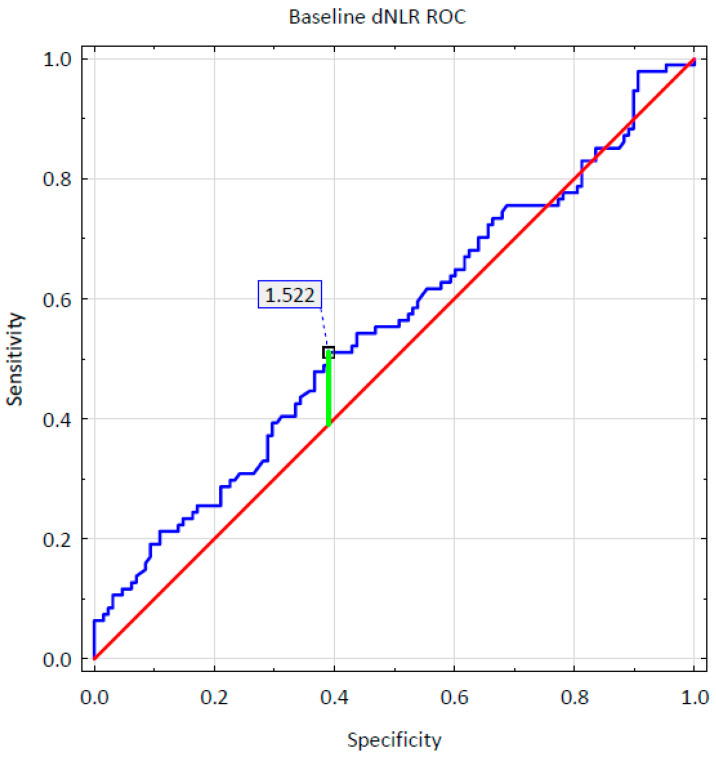
Baseline dNLR ROC curve.

The hematological parameters and calculated non-specific inflammatory markers were analyzed with a multinomial logistic regression. It was confirmed that a thousand per microliter (1000/µL) higher initial levels of neutrophils and lymphocytes were associated with, respectively, a 5.2-fold (OR = 5.201; 95% CI: 1.660–16.295; *p* = 0.005) and 4.2-fold (OR = 4.150; 95% CI: 1.135–15.182; *p* = 0.031) greater chance of achieving a super-response after a four-month-long therapy. Simultaneously, a thousand/microliter (1000/µL) higher total level of leukocytes was related to a fourfold (OR = 0.237; 95% CI: 0.081–0.691; *p* = 0.008) lower chance of reaching a super-responder status. Moreover, aderived Neutrophil-to-Lymphocyte Ratio (dNLR) value higher by one unit prior to treatment initiation was associated with a one and a half times higher chance of achieving a super-response regardless of the monoclonal antibody therapy applied (OR = 1.563; 95% CI: 1.053–2.321; *p* = 0.027). The Receiver Operating Characteristic (ROC) curve analysis was additionally conducted to evaluate the predictive ability of baseline dNLR. The ROC curve for baseline dNLR is shown in Figure 1. The ROC analysis determined a baseline dNLR cut-off value for a achieving a super response as 1.522 (95% CI: 0.794–1.978) with an Area Under the ROC curve (AUC) of 0.552 (95% CI: 0.474–0.629, *p* = 0.1891), sensitivity of 0.5106, and specificity of 0.3906. However, the obtained AUC value showed that the role of dNLR as an independent predictor was limited and not statistically significant due to other parameters affecting the predictive model.

**Figure 2:**
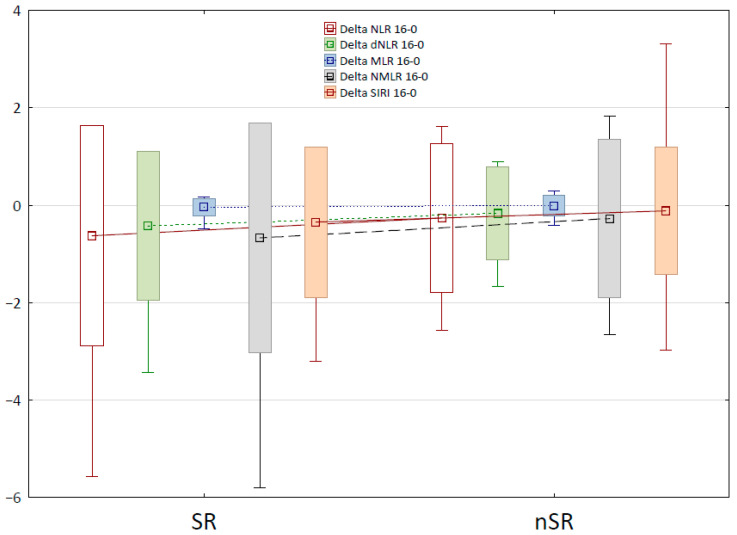
The box-and-whisker plot for changes in the selected Blood Count-Derived Inflammatory Markers between week 0 and 16 of the therapy.

Using the Mann–Whitney U-test, changes over time in blood-count-derived inflammatory biomarkers were also calculated and compared between the SR and nSR groups. Table 4 presents the definitions and changes in the selected markers for which the differences between SR and nSR groups were statistically significant, between therapy weeks 0 and 16. The box-and-whisker plot for these markers is shown in Figure 2. The decrease in values of Neutrophil-to-Lymphocyte Ratio (NLR), dNLR, Monocyte-to-Lymphocyte Ratio (MLR), Neutrophil-to-Monocyte-to-Lymphocyte Ratio (NMLR), and Systemic Inflammation Response Index (SIRI) biomarkers during biological treatment was statistically significantly higher in the group of super-responders than in the nSR group, which confirms a substantially better response to therapy. A reduction in Aggregate Index of Systemic Inflammation (AISI), Systemic Immune-Inflammation Index (SII), Platelet-to-Lymphocyte Ratio (PLR), and NMR (Neutrophil-to-Monocyte Ratio) parameters over time was also observed; however, no statistically significant disparities were identified between the SR and nSR groups.

## 3. Discussion

Achieving a super-response is currently becoming the most sought-after aim in the treatment of psoriasis. Factors that determine or increase the likelihood of this response are not fully recognized and require further research. Moreover, studies conducted so far vary in terms of the definition of super-response and study groups, which leads to inconsistent results and conclusions. It seems that younger patients more frequently achieve a super-response [6,14,15,16,17] and are characterized by shorter disease duration [8,14,18,19], lower body weight [8,9,15], and Body Mass Index (BMI) value [6,8,9,14,16,17]. They are also presumably distinguished by a lower baseline severity of skin lesions [8,11,14,15,16] and are more often bio-naïve [14,16,18,19,20,21,22]. On the other hand, the aforementioned profile has not been confirmed in a series of research [7,23,24,25,26], and the results of the abovementioned studies are contradictory in terms of different variables. Table 5 presents factors affecting the likelihood of achieving a super-response and findings of various studies.

Our study found that older patients had a lower chance of achieving a super-response. No statistically significant correlations were identified between sex, body weight, BMI value, age of disease onset, disease duration before biological treatment initiation, the number of different biological drugs received, baseline severity of the skin lesions, and achieving a super-response. However, a relationship between a longer duration of methotrexate therapy and a higher chance of reaching a super-responder status was observed. This result should be interpreted with caution due to differences in the doses applied, routes of drug administration, and the time between discontinuation of MTX-therapy and initiation of biological treatment. No evidence was found on any influence of other previously applied conventional systemic therapies or phototherapy and their duration on the chance of achieving a super-response by patients undergoing biological treatment. Other studies have also reported a lack of impact of previously used systemic non-biological treatments [15,20,24,26]. Nevertheless, two analyses have indicated a higher likelihood of achieving a super-response by patients who received fewer systemic treatment methods prior to biological treatment; however, this observation was not statistically significant [7,15]. To the best of our knowledge, no other studies have assessed the effect of the duration of previously administered systemic non-biologic therapies and phototherapy on achieving a super-responder status.

The type of administered biological drug also has a substantial impact on the clinical response. Our study confirmed that, as compared to tildrakizumab, ixekizumab and bimekizumab therapies were associated with a 14-fold and 105-fold higher odds of achieving a super-response, respectively. Mastorino et al. also noticed that patients treated with IL-17 inhibitors, ixekizumab and brodalumab achieved a super-response more frequently [6]. Similar findings were reported by Morariu et al. [27] and Liu et al. [17], who observed the highest percentage of patients receiving ixekizumab in the SR group. An analysis involving IL-23 inhibitors exclusively showed that patients treated with risankizumab had a statistically significantly higher chance of achieving a super-response compared to those receiving tildrazumab therapy. Similarly, in our study group, among individuals treated with IL-23 inhibitors, the highest percentage of SR was observed for risankizumab (SR: 39.44% vs. nSR: 60.56%), and the lowest for tildrakizumab (SR: 9.09% vs. nSR: 90.91%).

Psoriatic skin lesions in special localizations, i.e., the scalp, nails, anogenital area, folds, palms, and soles, are difficult to treat and significantly affect the patient’s quality of life. Esposito et al. noticed that the scalp and anogenital involvement was more frequently associated with a super-response, while nail and palmoplantar psoriasis were more demanding to treat [20]. According to a study conducted by Gargiulo et al., the non-occurrence of psoriatic changes in special localizations was associated with a higher likelihood of achieving a super-response by patients receiving anti-IL-23 monoclonal antibodies [19]. Some research suggests that nail involvement is a predictive factor indicating a low chance of reaching a super-response [12]. Analysis performed in our study group revealed that psoriatic skin lesions on the palms and soles were associated with a fivefold lower chance of achieving a super-response during biological therapy.

Psoriasis, an immunologically mediated chronic inflammatory disease, promotes co-occurrence of various metabolic diseases, mental disorders, inflammatory bowel disease, or skin diseases [1,2,3,4,28]. For this reason, when selecting a proper treatment strategy all comorbidities, including psoriatic arthritis, need to be considered. It appears that patients with fewer comorbidities have a higher likelihood of achieving a super-response during biological therapy [9,17,20,21,24]. Some studies suggest that the co-occurrence of PsA reduces the chance of reaching the most favorable response to treatment [17,20]; however, this relationship has not been confirmed in other studies [6,9,15,16,19,23]. The results of our study do not prove the influence of PsA on achieving a super-response in terms of skin lesions. Nevertheless, our findings show that the comorbidity of non-alcoholic fatty liver disease decreases the chance of a super-response by over two times. This outcome is consistent with previous studies [17] and the pathogeneses underlying both diseases. In a meta-analysis involving 109,806 participants, it was established that patients with moderate-to-severe psoriasis had a 4.01-times higher chance of metabolic dysfunction-associated steatotic liver disease (MAFLD) compared to non-psoriatic individuals [29]. Furthermore, a greater risk of cirrhosis was reported in individuals with psoriasis and psoriatic arthritis, and the risk of liver disorders grew with the increase in the body surface area affected by psoriasis [30]. The hepatodermal axis is increasingly acknowledged due to the common pathogenesis and mutual exacerbation of both disease entities. Adipose tissue is a source of various proinflammatory cytokines, including adiponectin, leptin, resistin, TNF-α, and IL-6, participating in the pathogenesis of both psoriasis and non-alcoholic fatty liver disease (NFLD). The excessive secretion of these proinflammatory cytokines in the course of psoriasis raises insulin resistance, which is a crucial factor underlying NFLD. Also, the progressing hepatic steatosis causes a further increase in insulin resistance, thus leading to a vicious circle. Moreover, a higher level of IL-17, a key cytokine in the pathogenesis of psoriasis, may be associated with a faster progression of MAFLD to steatohepatitis and even to hepatocellular carcinoma [31].

The importance of genetic background in the pathogenesis of psoriasis is commonly known [5,32]. Liu et al. observed a statistically significantly more frequent family history of psoriasis in a group of SR [17]. Nevertheless, findings obtained in other research do not support this outcome [8,12,23]. In our study, positive family history was also more frequently reported in the SR group; however, this relationship was not statistically confirmed, probably due to the heterogeneity of the study group and the multifactorial pattern of psoriasis inheritance. The source literature provides data on the impact of genetic background on the effectiveness of various biological drugs. It seems that patients carrying HLA-C*06:02/HLA-C*04 alleles have a higher likelihood of achieving a positive clinical response during the ustekinumab therapy [11,33]. Moreover, a relationship was observed between certain polymorphisms and the results of the treatment with anti-TNF drugs [34]. In a study conducted on patients receiving brodalumab, rs495337 (SPATA2), rs6311 (HTR2A), and rs4085613 (LCE3D) polymorphisms were associated with a positive response to the treatment at month 12 of the therapy [35]. The occurrence of eight specific single nucleotide polymorphisms (SNPs) indicates a promising response to sekukinumab therapy. The rs34085293 (DDX58_v1) and rs2304255 (TYK2_v3) SNP variants are in particular related to achieving a super-response in the treatment with the aforementioned IL-17A inhibitor [10]. However, further research is required to determine the genetic profile of patients who develop the most favorable response to the therapy with certain biological drugs.

Regardless of the patient’s genetic profile, it would be beneficial to distinguish between widely available biomarkers that could enable personalization of treatment by selecting the most suitable biological therapy with the highest likelihood of achieving a super-response in the patient. Ziolkowska-Banasik et al. suggested an IL-18/IL-13 serum level ratio as a super-response predictive factor for secukinumab therapy [26]. In another prospective study, it was confirmed that individuals presenting a positive response to the treatment with TNF-α inhibitors were characterized by a lower baseline NLR value compared to non-responders. In patients undergoing adalimumab therapy, lower baseline IL-6 levels were additionally reported. No such correlations were observed in the groups receiving IL-23, IL-12/23, IL-17A, and Interleukin-17 receptor (IL-17R) inhibitors [36].

The literature provides examples of the use of non-specific blood count-derived inflammatory markers as predictive factors in numerous disease entities [37,38,39]. It has been confirmed that biological treatment may cause a decrease in levels of inflammatory markers in the course of psoriasis [40,41,42]. Different markers, i.e., AISI, SIRI, SII, PLR, NLR, dNLR, and MLR, have been positively correlated with the severity of psoriatic skin lesions; however, dNLR appears to be the most reliable one in terms of skin lesion severity among all analyzed blood count-derived inflammatory markers [43]. Our study confirmed that a higher baseline dNLR value was associated with a statistically significantly greater chance of achieving a super-response.However, the AUS value obtained in ROC analysis showed that the role of dNLR as an independent predictor was limited. To the best of our knowledge, there is only one other study that assessed dNLR as a predictive biomarker of response to biological treatment of psoriasis. Morariu et al. reported that achieving a super-responder status was related to higher dNLR and SIRI values at baseline [27].

The main limitations of our study were its retrospective nature and relatively small groups of patients treated with individual biological drugs. Among patients receiving TNF-α inhibitors, the study group included only those undergoing adalimumab and infliximab therapies. In particular, the groups of patients treated with infliximab and ustekinumab were small, which makes it difficult to draw strong conclusions. Another limitation of our study was its single-center nature. A larger, multicenter, prospective study should be conducted to assess the suitability of a dNLR as a predictive biomarker of a super-response in the therapy of psoriasis with individual biological drugs. Moreover, a validation should be performed to establish reference ranges indicating a positive response to biological treatment.

## 4. Materials and Methods

Our single-center retrospective study involved patients suffering from psoriasis and receiving biological therapy with IL-12/23 (ustekinumab), IL-23 (guselkumab, risankizumab, tyldrakizumab), IL-17A (ixekizumab, sekukinumab), IL-17A/F (bimekizumab), or TNF-α (adalimumab, infliximab) inhibitors in the Department of Dermatology and Venereology, Medical University of Lodz, in the period from 1 March 2015 to 1 March 2025.

The research included patients with diagnosed moderate-to-severe plaque psoriasis (patients with psoriatic lesions severity on the PASI scale > 10 and/or BSA > 10% and/or DLQI > 10 points) who were unsuccessfully treated with at least two conventional methods (methotrexate, ciclosporin, acitretin, PUVA therapy), had contraindications to conventional systemic treatment, or developed adverse reactions during these therapies. The only exception were patients aged under 18 who qualified for biological treatment directly after an unsuccessful topical therapy.

The Psoriasis Area and Severity Index (range from 0 to 72; a higher score is associated with more severe psoriasis), the Body Surface Area scale (range from 0 to 100% of the body surface area affected by psoriatic lesions) and the Dermatology Life Quality Index (range from 0 to 30; a higher value is associated with a lower quality of life) were used to assess the severity of psoriasis.

The following exclusion criteria were defined for this study: active infection at the time of laboratory testing, systemic steroid therapy, pregnancy and lactation period, diagnosed other skin diseases with an inflammatory background, simultaneous treatment with a biological drug and any conventional method, and nail involvement without skin lesions. Patients receiving biological drugs for less than 12 weeks and with lacking data were also excluded from this research. The super-response in our analysis was defined as achieving PASI100 at week 16 (±4 weeks) of therapy. Primary and secondary treatment failures were characterized accordingly to the B.47 drug program financed by the Ministry of Health of the Republic of Poland. A primary failure meant that the patient did not reach at least a 75% reduction in the Psoriasis Area and Severity Index (PASI75) or a 50% reduction in the Psoriasis Area and Severity Index (PASI50) with a simultaneous decrease in the DLQI (Dermatology Life Quality Index) or cDLQI (Children’s Dermatology Life Quality Index) score by at least five points at week 16 (±4 weeks) of treatment. A secondary failure was defined as an increase in the severity of the disease characterized by PASI > 10, BSA > 10% (Body Surface Area), and DLQI > 10 points during two consecutive follow-up appointments.

This retrospective study relied exclusively on medical histories of patients including the severity of psoriatic skin lesions, treatment of psoriasis, anthropometric data, comorbidities, family medical history of psoriasis, and the results of laboratory tests. Peripheral venous blood samples required for laboratory tests were collected from patients in a sitting position before the therapy initiation and at week 16 (±4) of treatment. Hematological inflammatory parameters were analyzed. Based on the results of laboratory tests, the levels of leukocytes, neutrophils, lymphocytes, monocytes and platelets were used to determine and analyze Systemic Immune-Inflammation Index (SII), Systemic Inflammation Response Index (SIRI), Aggregate Index of Systemic Inflammation (AISI), Neutrophil-to-Lymphocyte Ratio (NLR), Platelet-to-Lymphocyte Ratio (PLR), Neutrophil-to-Monocyte Ratio (NMR), derived Neutrophil-to-Lymphocyte Ratio (dNLR), Neutrophil-to-Monocyte-to-Lymphocyte Ratio (NMLR), Monocyte-to-Lymphocyte Ratio (MLR) at both time points—at week 0 and 16 (±4 weeks).

This research was conducted in accordance with the Declaration of Helsinki, Good Clinical Practice rules, and all applicable legal regulations. The patients were provided with psoriasis treatment in line with recommendations of medical associations and clinical indications. The study obtained a positive opinion from the Bioethics Committee at the Medical University of Lodz (decision No. RNN/226/25/KE).

### Statistical Analysis

The patients were divided into two groups, being assigned either to super-responders or non-super-responders, according to the observed clinical response to therapy. The study aimed to identify and assess clinical and demographic differences between these groups and to determine a potential biomarker for a high likelihood of achieving a super-response by a particular patient. The analysis used widely available blood-count-derived inflammatory markers, i.e., SII, SIRI, AISI, NLR, PLR, NMR, dNLR, NMLR, and MLR. In order to identify the aforementioned possible predictor, biomarkers based on the complete blood count were calculated at week 0 and 16 (±4 weeks) and compared between the groups.

The statistical analysis of the data was conducted using Statistica 13.3 analytics software. Pearson’s chi-squared or Fisher’s exact tests were used, depending on the data, to determine associations between the qualitative variables. To assess factors associated with belonging to the SR group, the multinomial logistic regression analysis was conducted. Changes over time for selected parameters evaluated at both time points were compared between the SR and nSR groups using the Mann–Whitney U-test. ROC analysis was used to identify the cut-off values. *p*-values < 0.05 were considered statistically significant.

## 5. Conclusions

The results of our study indicate that individuals who have a higher chance of achieving a super-response are younger patients, with no psoriatic lesions on the palms and soles, not suffering from non-alcoholic fatty liver disease, previously treated with methotrexate, and characterized by a higher level of dNLR at the beginning of treatment. Hematological inflammatory parameters are widely available and inexpensive, and for that reason appear to be perfect biomarkers that enable an early treatment personalization by selecting the most suitable therapy. Further research is required to assess the dNLR biomarker in this respect.

## Figures and Tables

**Table 1:** Clinical data of the study group.

	Mean Value	Median	Minimum	Maximum	Standard Deviation
Age of treatment initiation (years)	44.00	42.00	10.00	79.00	14.74
PASI-0	17.57	18.60	0.00	50.00	8.16
BSA-0 [%]	23.43	21.00	0.00	97.00	14.42
DLQI-0 [pts]	20.52	21.00	0.00	31.00	5.55
PASI-16	1.90	0.40	0.00	24.00	3.73
BSA-16 [%]	2.84	0.20	0.00	28.00	5.35
DLQI-16 [pts]	2.69	0.00	0.00	25.00	4.99
PASI-40	2.00	0.00	0.00	24.00	4.23
BSA-40 [%]	2.77	0.00	0.00	47.00	6.89
DLQI-40 [pts]	2.41	0.00	0.00	26.00	5.10
Duration of biological treatment [in months]	21.79	18.00	3.00	118.00	16.53
AE	0.14	0.00	0.00	2.00	0.41
Number of previously used biological drugs	0.45	0.00	0.00	5.00	0.82
Weight [kg]	86.13	85.00	35.00	155.00	21.65
Height [cm]	171.99	172.50	115.00	193.00	10.50
BMI [kg/m^2^]	29.09	28.30	16.00	87.00	7.35

PASI-0/BSA-0/DLQI-0–PASI/BSA/DLQI score before the treatment initiation; PASI-16/BSA-16/DLQI-16–PASI/BSA/DLQI score at week 16 (±4) of biological treatment; PASI-40/BSA-40/DLQI-40–PASI/BSA/DLQI score at week 40 (±4) of biological treatment; AE—number of reported adverse reactions.

**Table 2:** Duration of treatment with respective conventional methods (in months).

	Mean Value	Median	Minimum	Maximum	Standard Deviation
Methotrexate	8.29	4.00	0.00	120.00	15.57
Ciclosporin	5.82	2.30	0.00	144.00	17.07
Acitretin	1.05	0.00	0.00	48.00	3.72
PUVA therapy	0.79	0.00	0.00	84.00	5.72
UVB-NB phototherapy	1.14	0.00	0.00	24.00	2.37

**Table 3:** Comorbidities in the study group.

	No. of TC with a Comorbidity	No. of TC with Available Data	Percentage
Psoriatic arthritis	55	222	24.77%
Nicotine addiction	50	221	22.62%
Diabetes melitus	37	222	16.67%
Hyperlipidemia	138	221	62.44%
Non-alcoholic fatty liver disease	45	221	20.36%
Depression/anxiety	38	221	17.19%
Hypertension	92	221	41.63%
Chronic kidney disease	13	222	5.86%
Ischemic heart disease	12	221	5.43%
Thyroid diseases (hypothyroidism, hyperthyroidism, thyroid nodules)	25	221	11.31%

**Table 4:** Definitions and changes in the selected Blood Count-Derived Inflammatory Markers between week 0 and 16 of the therapy, *p*-value for Mann–Whitney U-test.

	Definition	Change Week 0 to 16 SR Group	Change Week 0 to 16 nSR Group	*p*-Value
NLR	neutrophils/lymphocytes ratio	−0.63 ± 1.13	−0.26 ± 0.76	0.0304
dNLR	neutrophils/(white blood cells − neutrophils)	−0.43 ± 0.77	−0.17 ± 0.48	0.0210
MLR	monocytes/lymphocytes ratio	−0.04 ± 0.09	−0.01 ± 0.11	0.0213
NMLR	(neutrophils + monocytes)/lymphocytes ratio	−0.67 ± 1.18	−0.28 ± 0.81	0.0216
SIRI	(neutrophils × monocytes)/ lymphocytes	−0.35 ± 0.77	−0.12 ± 0.65	0.0453

**Table 5:** Factors affecting the likelihood of achieving a super-response and findings of various studies.

Research	Biological Drugs	Research Type	Patients	Super-Response Definition	Selected Clinical Factors Increasing the Likelihood of Achieving a Super-Response
Schäkel K et al. [14]	Guselkumab	Clinical trial	All	PASI100 at weeks 20 and 28	Younger age, BMI < 25, shorter disease duration, bio-naïve status
Reich K et al. [15]	Guselkumab	Clinical trial	All	PASI100 at weeks 20 and 28	Younger age, lower body weight, lower baseline IGA and PASI score
Gerdes S et al. [18]	Guselkumab	Real world	All	PASI100 at weeks 20 and 28	Shorter disease duration, bio-naïve status
Mortato E et al. [16]	Guselkumab	Real world	All	PASI100 at week 20	BMI < 30, lower baseline PASI, bio-naïve status
Ruiz-Villaverde R et al. [23]	Guselkumab	Real world	All	PASI100 at weeks 12 and 24	No depression, low DLQI and Visual Analogic Scale of Pruritus score
Gargiulo L et al. [19]	Risankizumab	Real world	All	PASI100 at week 52	Shorter disease duration, no cardiometabolic diseases, bio-naïve status, no involvement of difficult-to-treat localizations
Loft N et al. [9]	TNF-α inhibitors, Ustekinumab	Real world	All	PASI < 3 skin lesion severity for a period of 6 to 60 months	Lower body weight, lower BMI, fewer comorbidities, lower baseline PASI score, no nicotinism, higher socioeconomic status
Mastorino L et al. [6]	Ixekizumab, Secukinumab, Brodalumab, Tildrakizumab, Guselkumab	Real world	Bio-naïve	PASI100 at weeks 16 and 28	Younger age, lower BMI, earlier onset of psoriasis, no obesity or diabetes, higher baseline PASI score, treatment with Il-17 inhibitors
Rompoti N et al. [7]	Brodalumab	Real world	All	PASI < 1 at weeks 12 and 16	No statistically significant factors were identified
Esposito M et al. [20]	Bimekizumab	Real world	All	PASI100 at weeks 4 and 16	Age ≤ 65, male gender, no comorbidities, baseline PASI score ≥ 10 and <20, bio-naïve status, no arthritis
Liu Y et al. [21]	Adalimumab	Real world	All	PASI100 at weeks 12 and PASI < 1 at weeks 24 or 32	Bio-naïve status (only for reaching PASI90), female gender, no comorbidities, higher HDL level
Liu Y et al. [17]	Ixekizumab, Secukinumab, Ustekinumab, Adalimumab, Guselkumab	Real world	Bio-naïve	PASI100 at week 4 and maintaining PASI < 1 to week 48	BMI < 25, fewer comorbidities, no arthritis, family history of psoriasis, lower triglyceride to high-density lipoprotein cholesterol ratio
Mason KJ et al. [24]	Guselkumab, Certolizumab, Golimumab, Onercept, Adalimumab, Ustekinumab, Etanercept, Secukinumab, Infliximab, Ixekizumab, Brodalumab, Efalizumab	Real Word	Bio-naïve	treatment with the first biological drug for ≥5 years in monotherapy	Male gender, fewer comorbidities, lower DLQI score at baseline, type of drug
Menéndez Sánchez M et al. [25]	Risankizumab, Guselkumab, Tildrakizumab	Real Word	All	PASI100 at weeks 16 and 24	Type of drug
Ziolkowska-Banasik D et al. [26]	Sekukinumab	Real world	Bio-naïve	PASI100 at week 12	Lower baseline monocyte count, Higher serum IL-13 concentration at baseline, Lower serum IL-18 concentration at baseline, Lower IL-18/IL-13 ratio, Lower IL-17/IL-13 ratio
Fratton Z et al. [12]	Bimekizumab	Real world	All	Early Super Response: PASI100 at week 4	Lower baseline PASI score, less than 3 biologic treatment failures, no psoriatic nail involvement
Feldman SR et al. [8]	Tildrakizumab	Clinical trial	All	PASI90-100 at week 28	Lower body weight, lower BMI, shorter disease duration
Marcelli L et al. [22]	Guselkumab	Real world	All	PASI100 at week 20	Bio-naïve status, co-occurrence of PsA
Morariu SH et al. [27]	Adalimumab, Etanercept, Infliximab, Certolizumab, Ixekizumab, Secukinumab, Tildrakizumab, Risankizumab, Guselkumab, Ustekinumab, Apremilast	Real world	Bio-naïve	PASI100 at week 24	Scalp involvement, higher baseline dNLR value

## Data Availability

The data presented in this study are available on request from the corresponding authors. The data are not publicly available due to privacy and ethical restrictions.

## Abbreviations

The following abbreviations are used in this manuscript:

TNF-α	Tumor Necrosis Factor-alpha
IL-17	Interleukin 17
IL-12	Interleukin 12
IL-23	Interleukin 23
Il-6	Interleukin 6
IL-18	Interleukin 18
IL-13	Interleukin 13
IL-17R	Receptor for interleukin 17
PsO	Psoriasis
UVB-NB	Narrowband Ultraviolet B
PUVA	Psoralen Ultra-Violet A
SR	Super-responders
nSR	Non-super-responders
PsA	Psoriatic arthritis
TC	Treatment cycles
BMI	Body Mass Index
MAFLD	Metabolic dysfunction-associated steatotic liver disease
NFLD	Non-alcoholic fatty liver disease
IL-17A	Subunit A of interleukin 17
IL-17AF	Subunit A and F of interleukin 17
IL-12/23	Interleukin 12 and 23
Anti-IL-17A	Antagonist of subunit A of interleukin 17
Anti-TNF	Tumor necrosis factor inhibitor
Anti-IL-23	Interleukin 23 antagonist
PASI	Psoriasis Area and Severity Index
PASI0	Baseline Psoriasis Area and Severity Index
PASI100	Complete clearance of psoriatic skin lesion
PASI90	90% reduction in psoriatic skin lesions
PASI75	75% reduction in psoriatic skin lesions
PASI50	50% reduction in psoriatic skin lesions
PASI16	Psoriasis Area and Severity Index score at week 16 (±4) of biological treatment
PASI40	Psoriasis Area and Severity Index score at week 40 (±4) of biological treatment
AE	Adverse reactions
BSA	Body Surface Area
BSA0	Baseline Body Surface Area
BSA16	Body Surface Area at week 16 (±4) of biological treatment
BSA40	Body Surface Area at week 40 (±4) of biological treatment
cDLQI	Children’s Dermatology Life Quality Index
DLQI	Dermatology Life Quality Index
DLQI0	Baseline Dermatology Life Quality Index score
DLQI16	Dermatology Life Quality Index score at week 16 (±4) of biological treatment
DLQI40	Dermatology Life Quality Index scoreat week 40 (±4) of biological treatment
SII	Systemic Immune-Inflammation Index
SIRI	Systemic Inflammation Response Index
AISI	Aggregate Index of Systemic Inflammation
NLR	Neutrophil-to-Lymphocyte ratio
PLR	Platelet-to-Lymphocyte ratio
NMR	Neutrophil-to-Monocyte ratio
dNLR	derived Neutrophil-to-Lymphocyte Ratio
NMLR	Neutrophil-to-Monocyte-to-Lymphocyte Ratio
MLR	Monocyte-to-Lymphocyte Ratio
SNPs	Single Nucleotide Polymorphisms
*p*	*p*-value
Cl	Confidence Interval
OR	Odds Ratio
